# Improvement of EGFR Testing over the Last Decade and Impact of Delaying TKI Initiation

**DOI:** 10.3390/curroncol28020102

**Published:** 2021-02-26

**Authors:** Félix Blanc-Durand, Marie Florescu, Mustapha Tehfe, Bertrand Routy, Raafat Alameddine, Danh Tran-Thanh, Normand Blais

**Affiliations:** 1 Hematology and Medical Oncology Service, Centre Hospitalier Universitaire de Montreal, University of Montreal, Montreal, QC H2X 3E4, Canada; felix.blancdurand@gmail.com (F.B.-D.); marie.florescu.chum@ssss.gouv.qc.ca (M.F.); mustapha.tehfe.chum@ssss.gouv.qc.ca (M.T.); bertrand.routy@umontreal.ca (B.R.); raafat.alameddine@gmail.com (R.A.); 2 Pathology Department, Centre Hospitalier Universitaire de Montreal, University of Montreal, Montreal, QC H2X 3E4, Canada; danh.tran-thanh.chum@ssss.gouv.qc.ca; 3 Hematology/Oncology Department, CHUM, 1000, Rue Saint-Denis, Montreal, QC H2X 0C1, Canada

**Keywords:** lung cancer, EGFR, real-world evidence, liquid biopsy and precision medicine

## Abstract

Background: Epidermal growth factor receptor (EGFR) is the most common oncogenic mutation in lung adenocarcinoma and tyrosine kinase inhibitors (TKIs) have been considered standard treatment for more than a decade. However, time to initiation of TKIs (TTIT) from diagnosis is often delayed and represents a challenge for clinicians. We aimed to assess the impact of TTIT on clinical outcomes and complications. Method: TTIT was defined as the time between confirmed advanced diagnosis and the initiation of a TKI. Complications during this pre-TKI period were retrospectively collected from all patients with EGFR-mutant non small cell lung cancer (NSCLC) in our institution. Results: 102 patients were diagnosed with EGFR mutated NSCLC between 2006 and 2019. The median PFS and OS were 12.9 and 22.5 months, respectively. TTIT was 5.7 months (95% CI 3.4–8) with a significant decrease in the latter years of this cohort. During the pre-TKI period, 23 patients received chemotherapy as first line treatment, of which 5 developed severe adverse events and 3 were not fit to receive TKI thereafter. Additionally, 29 patients had rapid clinical deterioration before initiation of first line TKI and 16 had to be hospitalized. Among the patients presenting a performance status deterioration, their prognosis was markedly affected compared to the remainder of the cohort (*p* = 0.01). Conclusion: Our real-world evidence study supports the concept that a delay to treat EGFR mutant NSCLC with TKIs is associated with adverse events, patient progression, hospitalization, and decreased overall survival. Rapid molecular diagnosis, including access to ctDNA technology may circumvent these deleterious delays.

## 1. Introduction

Over the last decades, significant improvements have been achieved in unraveling the molecular pathogenesis of lung adenocarcinoma. The discovery of molecular driver mutations, such as occurs in the epidermal growth factor receptor (EFGR) and anaplastic lymphoma kinase (ALK) genes have led to the development of targeted treatment strategies which have improved the outcomes of patients harboring these mutations [1].

EGFR-related lung cancer is more frequent in women, non-smokers [2] and patients of East Asian descent [3] and is found in 15% of patients with adenocarcinoma in North America [4]. Following the pivotal phase III randomized trials IPASS and EURTAC, first generation EGFR tyrosine kinase inhibitors (TKIs), gefitinib and erlotinib, demonstrated their superiority in terms of progression-free survival, compared with platinum-based chemotherapy and have been widely adopted as first line treatment NSCLC with EGFR mutations [5,6,7]. The second generation TKIs, afatinib and dacomitinib, have demonstrated improvements in PFS compared to first generation TKIs but with a significant toxicity profile that has limited their use [8,9]. More recently, the FLAURA phase III clinical trial compared first line osimertinib versus first generation TKI (gefitinib or erlotinib) and demonstrated longer OS and longer freedom from progression in the central nervous system with osimertinib [10,11]. Upon progression after optimal TKI treatment, immune checkpoint inhibitors provide responses in less than 5% of patients [12] therefore, several approaches including novel EGFR targeted therapies, combinations with chemotherapy, or antiangiogenics are undergoing active investigation [13,14,15,16].

These targeted treatments are usually associated with rapid tumor response within the first months of treatment [17]. Therefore, EGFR mutation testing is essential [18] and should be performed for patients with advanced non-squamous NSCLC, irrespective of the clinicopathologic features or when an adenocarcinoma component cannot be excluded [19].

Classically, after clinical and radiological suspicion of a lung neoplasm, the patient undergoes a tumor biopsy to confirm histology or obtain tissue diagnosis as part of a planned or urgent surgical procedure. EGFR testing needs a good quality sample with enough tumor cells and thus, may require multiple attempts to be conclusive. This multiple step strategy can lead to significant delays before the initiation of anti-EGFR TKIs and may expose patients to symptomatic progression and clinical deterioration.

Our institution is a large university hospital that has implemented EGFR testing in 2006 and officially started doing reflex testing for all advanced non-squamous NSCLC at the end of 2017. Initially, the analysis was performed when asked by the treating physician and progressively became reflexive in the pathology department for all patients with advanced non-squamous NSCLC. Similarly, in locally advanced diseases, the analysis was initially performed only at the time of metastatic relapse, but is now performed routinely at diagnosis [20,21].

The most frequent mutations described in NSCLC are localized on exon 19 or exon 21 and are associated with high sensitivity to anti-EGFR TKis [22]. However, rare mutations occurring in about 10% of patients localized on exon 18 and 20 have been described and display a variable sensitivity profile to currently available targeted treatments [23]. Our molecular platform originally screened only exon 19 and 21 mutations by in-house PCR. Since mid-2017, we also covered exon 18 and 20 with the commercial kit (“EGFR Mutation Analysis Kit”) from Entrogen^®^ company. Finally, since 2018, we have prioritized Illumina’s integrated next-generation sequencing platform MiSeq and use the Entrogen kit^®^ for poor quality samples.

Time to initiation of anti-EGFR TKIs (TTIT) is associated with several challenges for clinicians as the patient might deteriorate quickly or develop new symptomatic metastases before any therapy can even be initiated. In a publicly-funded health care system, rapid access to diagnostic procedures is often limited by many logistical factors such as access to interventional procedures, delays in pathology processing, and access to private insurance-covered medications. In order to better evaluate this impact, we aim to measure the TTIT of EGFR TKIs and the clinical consequences of delaying TKIs. Various studies have reported improved outcomes among patients with shorter wait times [24,25] although data specific to the EGFR mutated population are scarce [26].

## 2. Methods

### 2.1. Population

Every patient with advanced NSCLC harboring EGFR mutations (exon 18, 19, 20, and 21) between May 2006 and April 2019 and treated in our institution was included. The recruitment was based on molecular records when the analysis was performed at the CHUM or by a review of all the patients who had received at least one EGFR TKI (gefitinib, erlotinib, afatinib or osimertinib). Moreover, only patients with primary or secondary metastatic disease were analyzed. Data were collected and anonymized after data extraction from the personal medical record. This specific project was approved by the CHUM research and ethics committee (ref: 19.184–MJB, 2020-8592)

### 2.2. Endpoints

**Figure 1:**
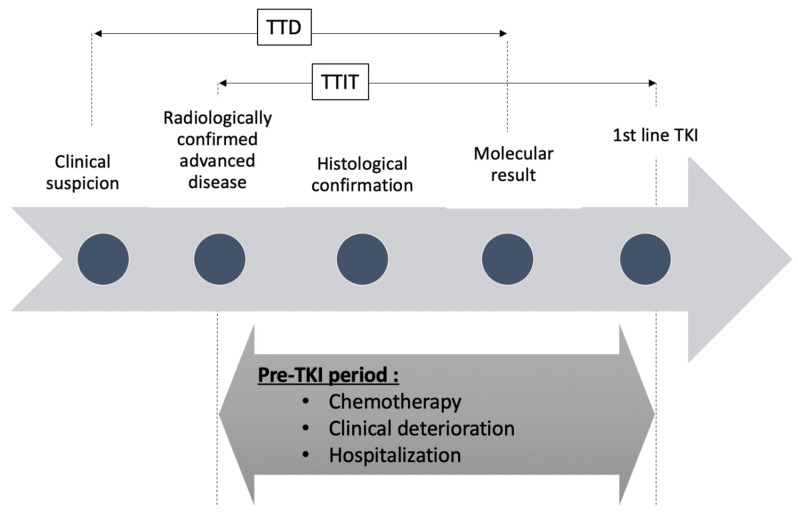
Timeline depicting pre- tyrosine kinase inhibitors (TKI) period for patients with epidermal growth factor receptor (EGFR) mutated NSCLC. TTD = time to detect EGFR mutation. TTIT: time to initiate TKI.

This retrospective, monocentric, real-world evidence study aimed to describe as a primary endpoint the time to initiation of EGFR-TKI (TTIT) defined as the time between radiologically confirmed advanced disease (scan (CT) or positron emission tomography scan describing distant metastasis or loco-regional recurrence) until initiation of a TKI (Figure 1).

We also assessed, as preplanned exploratory analysis, the time between clinical/radiological suspicion of lung cancer (loco-regional or advanced disease) and detection of EGFR mutation by pathology (time to detection: TTD) and the time to begin TKI (between molecular confirmation of EGFR mutation and first day of EGFR TKI). It should be noted that for primary metastatic diseases, the date of clinical/radiological suspicion and the date of radiologically confirmed advanced diseases are the same (Figure 1).

In addition, secondary objectives included the description of complications occurring during the period before initiating a TKI, overall survival (OS) from the date of histological diagnosis of lung cancer until death or loss of follow-up, progression-free survival with TKI therapy (PFS-TKI) from the first day of TKI until the date of progression (Response Evaluation Criteria in Solid Tumors (RECIST) 1.17; investigator assessment) or death.

Finally, post-hoc objectives included the analysis of OS with respect to the presence or absence of symptomatic disease, performance status deterioration (worsening of Eastern Cooperative Oncology Group [ECOG] status ≥1), and hospitalizations before initiation of a TKI.

### 2.3. Statistical Analysis

Graphical and statistical analyses were performed using Prism version 7.0a (GraphPad Software). Kaplan-Meier curve analysis and log-rank tests were performed to reveal the impact of the different mutations, the treatment modalities, and several clinical variables on survival. *p* values between the different periods of treatment were determined using a non parametric unpaired *t*-test.

## 3. Results

There were 427 patient files reviewed, 198 extracted from in-house pharmacy records and 229 out of pathological records. We identified a total of 102 patients who were diagnosed and treated for EGFR mutated advanced NSCLC between 2006 and 2019.

Clinical characteristics are summarized in Table 1. There were 70 (68.6%) women and the median age at diagnosis was 62.1 years (range 22 to 92). As expected, most patients were never smokers (52.9%) or light smokers (mean 22 pack-years) and the histological subtype was almost exclusively adenocarcinomas (96%). There were 45 (44.1%) patients with EGFR exon 19 deletion, 46 (45.1%) with the exon 21 L858R mutation, 3 (2.9%) patients were exon 18 mutated and 7 (6.9%) had exon 20 aberrations. There were 66 patients (64.7%) who presented with primary metastatic disease and harbored the same clinical characteristics as the global population outside of stage at first diagnosis.

Out of 102 patients, 72 (71%) received a TKI as 1st line treatment. In this group, 52% of patients received gefitinib, 19.6% afatinib, 15.7% erlotinib, and 3% osimertinib. Another 30 (30%) received either platinum-based chemotherapy (23 patients) or best supportive care (7 patients). After chemotherapy, 20 patients out of 23 received a TKI at relapse.

### 3.1. General Outcomes

Median time between confirmed advanced disease and the first day of anti-EGFR TKI (TTIT) was 174 days (95% CI 103–242 days), with a range from 1 to 2581 days. However, great improvement in TTIT was observed between quartiles: 2006–2010 period (402 days), 2011–2013 (199 days), 2014–2016 (148 days), and 2017–2019 (81 days), *p* < 0.001 (Table 2).

Median time to detection of an EGFR mutation (TTD) was 342 days in the whole population. This delay can be explained partly by patients with locally advanced disease for whom the EGFR analysis was performed only at relapse. Indeed, for primary metastatic disease, TTD was shorter (140 days) but still improved over time. However, as soon as the EGFR result was available, the time to begin a TKI was very short (6 days).

Out of the 66 primary metastatic patients, 29 (44%) underwent more than one procedure to obtain the histological confirmation (14 patients) or the molecular result (8 patients) and these situations were responsible for delays in the initiation of therapy.

### 3.2. Interval before the Initiation of a TKI

There were 23 patients (22.5%) who received platinum-based chemotherapy as first line treatment, mostly when EGFR results were not available (Table 3). Out of them, 5 developed severe adverse events related to the treatment, such as kidney failure, fatigue and nausea, moreover, 3 of these patients with severe side-effects did not go on to receive an EGFR TKI.

With improvement in diagnostic delays, the number of patients who received chemotherapy while awaiting EGFR results tended to decrease. Nevertheless, we observed an increase in this number in the last years (2017–2019) that is explained by the beginning of screening for exon 18 and exon 20 mutations in this time period and the preferential use of chemotherapy in some of these patients.

Clinical progression in patients before the initiation of a TKI led to many adverse outcomes. From the total group of 102 patients, 29 patients (28%) developed new symptoms, even despite the initiation of first line platinum-based chemotherapy (for 12 out of the 29 patients). This deterioration occurred mostly because of rapid disease progression (20 patients) and was associated with PS deterioration for 17 patients. Patients complained about fatigue (12 patients), chest pain (6 patients), dyspnea (8 patients), and cough (1 patient), secondary to pleural or pericardial effusion in 7 cases.

Out of them, 16 patients had to be hospitalized (duration range from 1 to 60 days), (Table 4), and most importantly, 2 deaths occurred before initiation of a TKI, one because of active tuberculosis, the other because of respiratory failure and bacterial pneumonia.

In all, a total of 10 patients (9.8%) did not receive any anti-EGFR treatment despite the existence of a mutation and, only one of them presented a rare “non-druggable” mutation (exon 20 insertion). Importantly, 3 of them because they presented major clinical deterioration during first line chemotherapy. There were 7 patients (6.9%) who were severely fragile and impaired at diagnosis and refused anti-EGFR TKI or presented rapid deterioration because of disease progression before the initiation of any treatment.

### 3.3. Survival Impact

In this real-world cohort not selected for treatment eligibility, median PFS and OS, calculated from the time of the diagnosis of advanced disease, were 12.9 and 22.5 months, respectively, which is consistent with the literature. The overall survival from initial histological diagnosis was 34.7 months (CI95% 25–40 m) (Supplementary Figure S1).

The median PFS while receiving TKI for patients who received targeted treatment as first line (mPFS = 12.3 months) or in second line after chemotherapy (mPFS = 13.1 months) was very similar (Supplementary Figure S2). We also did not find any OS or PFS difference between the type of EGFR TKI.

Median PFS on TKI therapy was statistically superior for exon 19 and exon 21 (17 and 16.4 months, respectively) compared to exon 18 and 20 (10.3 months, *p* = 0.02).

**Figure 2:**
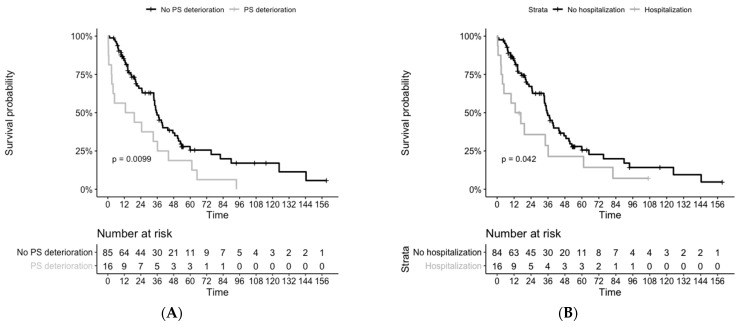
Overall survival from histological diagnosis, depending on pre-TKI period complications: (**A**) with or without performance status deterioration, 1 patient with missing survival data (**B**) with or without any hospitalization. PS = performance status.

For those patients who had a deterioration in performance status pre-TKI, we observed decreased survival (n = 16, mOS = 35.8 months versus 16.1 months, *p* = 0.009), as well as in those who required pre-TKI hospitalization (n = 16, mOS = 35.3 months versus 14.8 months, *p* = 0.04), (Figure 2). However, we didn’t notice any difference between the two groups TTIT and TTD. Patients with symptomatic disease had numerically lower survival rates (n = 20, mOS = 34.8 months versus 19.3 months, *p* = 0.5).

## 4. Discussion

The time to diagnosis and treatment of EGFR mutated NSCLC can be prolonged by several months and this delay exposes patients and treating physicians to several challenges. Before the initiation of TKI therapy, many patients develop clinical deterioration because of rapid disease progression and cannot receive optimal treatment. In our cohort, this was the case for 10 patients out of 102. Moreover, 29 patients (28%) with clinical deterioration had significant consequences such as hospitalization and pleural drainage before the initiation of TKI therapy. We demonstrated that these situations are associated with a worse prognosis. These complications could be prevented by an earlier molecular diagnosis. Earlier diagnosis would be expected to be associated with an improved quality of life for these patients and have the potential for decreased hospitalization rates and cost savings.

In our study, we present evidence that patients who received cytotoxic chemotherapy as first line systemic treatment exhibited sustained sensitivity to EGFR-TKIs at relapse. Most of them had rapid disease progression at onset with severe symptoms and were initiated on chemotherapy for a few cycles and then were switched to an EGFR TKI at molecular confirmation. Thus, upfront chemotherapy can provide a bridge to TKI therapy for certain patients with aggressive disease or when there is difficulty to obtain a biopsy or a molecular profile of the tumor, for example, in instances of brain only metastases or challenging situations where a biopsy is not feasible or has failed to provide an adequate sample. This situation can now be curtailed in many patients by the use of ctDNA technology very early in the diagnostic pathway. Our results suggest that patients treated with upfront chemotherapy conserve sensitivity to TKI therapy when used as a post-chemotherapy treatment. Nonetheless, a substantial portion of patients don’t receive any systemic treatment after first chemotherapy, as shown in our real-world cohort. In light of the recent demonstration of an overall survival benefit and the benefit in terms of increased activity in the central nervous system (compared to chemotherapy and other TKIs) of osimertinib in the first-line setting however, every effort should be made to accelerate the availability of the molecular sub-type of lung cancer [11].

Rare mutations affecting exon 18 and 20 were detected in 10.8% of our cohort but this number is underestimated since they were screened only during the latter years of our analysis. As shown by others, they are associated with variable outcomes with EGFR targeted treatment and thus, may benefit more from second/third generation TKIs, platinum-based chemotherapy, or novel approaches, depending on the specific mutation. In large retrospective cohorts already published, patients with uncommon EGFR mutated NSCLC presented significantly lower mPFS (from 3 to 9 months) compared to patients with exon 19 and 21 mutations [27,28,29,30]. However, there was a survival benefit for patients who received a TKI anytime during the course of their disease versus those who never received a TKI, suggesting the importance of these treatments, at least for some of these patients [27]. This may be more relevant to point mutations compared to exon 20 insertions. Indeed, afatinib has been approved specifically for three atypical EGFR mutations: G719X, S768I, and L861Q mutations based on some efficacy for this subgroup [31,32]. Otherwise, preliminary data are suggesting favorable activity of osimertinib in patients harboring these uncommon mutations [33]. On the contrary, exon 20 insertions are associated with de novo resistance to first and second generation EGFR TKIs and poor prognosis, therefore, in this situation, many physicians still consider that platinum-based chemotherapy is the standard option [34,35].

Patients with performance status deterioration and symptomatic disease leading to hospitalization during investigation are frequently seen and have markedly worse outcomes. Even though these situations are markers of aggressive cancers with poor inherent prognosis, we strongly believe that rapid treatment of these patients may prevent complications and improve their survival. When the EGFR result was available, time to begin the anti-EGFR TKI was very short (6 days). We thus conclude from our analysis that we need to focus our efforts on the delay to obtain molecular testing results. During the last decade, liquid biopsies to detect EGFR mutations in circulating tumor DNA (ctDNA) have been developed and demonstrate good sensitivity (between 62 and 75%) and specificity (between 79 and 96%) [36,37]. In a real-world setting, a large trial concluded that ctDNA is feasible with an excellent level of concordance with tissue diagnosis [38]. In expert academic centers, the turnaround time of ctDNA testing was 3 days compared to 12 days for tissue testing [39]. In a real-life setting, tissue testing delays are expected to be longer, mostly related to the logistics of obtaining the tissue sample, as shown by our study.

## 5. Conclusions

Our analysis of this real-world cohort suggests that earlier access to a molecular diagnosis of EGFR-related lung cancer is expected to decrease complications and hospitalizations related to delays in initiation of therapy and avoid early deaths. Indeed, we observed that around 10% of our patients were not candidates to receive any life-prolonging targeted treatment because of several complications in the pre-treatment period. These results support early and rapid diagnostic assays such as liquid biopsies and efforts to streamline biopsies and the molecular diagnosis in tissue.

## Figures and Tables

**Table 1:** Clinical characteristics of the whole population and the primary metastatic diseases.

Clinical Characteristics	N = 102
**Age-Yr**	62.1 (21.8–91.5)
**Sex**	
Male	32 (31.4%)
Female	70 (68.6%)
**Smoking status**	
Current/former	44 (43.1%)
Never/light	54 (52.9%)
Unknown	4 (3.9%)
**Stage at diagnosis**	
I-II	19 (18.6%)
IIIA/C	16 (15.7%)
IV	66 (64.7%)
unknown	1 (1%)
**Histologic subtype**	
Adenocarcinoma	98 (96.1%)
Squamous	2 (1.9%)
Other	2 (1.9%)
**EGFR mutation**	
Exon 19	45 (44.1%)
Exon 21	46 (45.1%)
Others	11 (10.8%)
Exon 18	7 (6.9%)
Exon 20	3 (2.9%)
Other	1 (1%)
**1st line TKI**	
Erlotinib	16 (15.7%)
Gefitinib	53 (52.0%)
Afatinib	20 (19.6%)
Osimertinib	3 (2.9%)
None	10 (9.8%)

**Table 2:** Time to histological confirmation, EGFR mutation result and 1st day of anti-EGFR TK. * Some patients had started TKIs before molecular diagnosis.

Parameters	Mean (Days)	Range (Days)
Time between 1st biopsy and histological confirmation	24.2	1–190
2006–2010 (n = 14)	31.5	3–147
2011–2013 (n = 28)	42.4	4–190
2014–2016 (n = 32)	18.2	1–72
2017–2019 (n = 28)	9.1	2–20
Time between clinical suspicion of cancer and EGFR result (TTD)	342.9	13–3003
2006–2010 (n = 14)	1730.3	85–3003
2011–2013 (n = 28)	438.6	13–2056
2014–2016 (n = 32)	187.6	14–1058
2017–2019 (n = 28)	48.4	17–119
For primary metastatic disease: Time between clinical suspicion and EGFR result	140.4	13–3003
2006–2010 (n = 2)	1542.8	85–3003
2011–2013 (n = 17)	190.6	13–1204
2014–2016 (n = 22)	78.7	14–451
2017–2019 (n = 25)	48.4	17–119
Time between confirmed advanced disease and 1st day TKI (TTIT)	173.6	1–2431
2006–2010 (n = 14)	402.3	40–2431
2011–2013 (n = 28)	199.7	1–1256
2014–2016 (n = 32)	148.2	2–1046
2017–2019 (n = 28)	81.7	9–250
For primary metastatic disease: Time between EGFR result and 1st day TKI	6.1	−1035–404

**Table 3:** 1st line treatment in the overall population.

1st Line Treatment	N = 102
Platinum-based chemotherapy	23 (22.5%)
2006–2010 (n = 14)	6 (42.9%)
2011–2013 (n = 28)	8 (28.6%)
2014–2016 (n = 32)	4 (12.5%)
2017–2019 (n = 28)	5 (17.8%)
TKI	71 (69.6%)
Other	1 (1%)
No treatment	7 (6.9%) (3 refused, 4 unfit)

**Table 4:** Complications reported during the pre-TKI period. N = 101, 1 patient with non-available data. PS = performance status.

Pre-TKI Complications		Number of Patients n = 101	Symptoms	Number of Patients	Duration Median (Range)
Clinical deterioration (n = 29)	Symptomatic disease	20	PS deterioration	17	
			Dyspnea	8	
			Pain	6	
			Cough	1	
			Pleural effusion	6	
			Pericardial effusion	1	
	Other causes	9	Chemotherapy severe adverse events	5	
			Anxiety	2	
			Pulmonary embolism	1	
			Tuberculosis	1	
Hospitalization		16	Pleural effusion	3	17 days (1–30)
			Fatigue	8	21 days (1–60)
			Dyspnea	3	17 days (1–30)
			Pain	3	1 day (1–21)
			Pulmonary embolism	1	3 days
			Tuberculosis	1	30 days

## Data Availability

Datasets generated for the current study are not publicly available considering confidentiality reasons. Anonymized data may be available from the corresponding author on justified request.

## Supplementary Materials

The following are available online at https://www.mdpi.com/1718-7729/28/2/102/s1, Figure S1: Overall survival from histological diagnosis in the whole population (N = 102), Figure S2: Progression-free survival (from the time of initiation of TKI therapy) in patients treated upfront with TKIs or in patients having received chemotherapy as a first line regimen.
