# Supplementary material for: Improvement of EGFR Testing over the Last Decade and Impact of Delaying TKI Initiation
Source: Curr Oncol. 2021 Feb 26;28(2):1045–55. doi: 10.3390/curroncol28020102 (PMC8025752; doi:10.3390/curroncol28020102)
Supplement: Supplementary file 1 [file curroncol-28-00102-s001.pdf]

Supplementary Figures

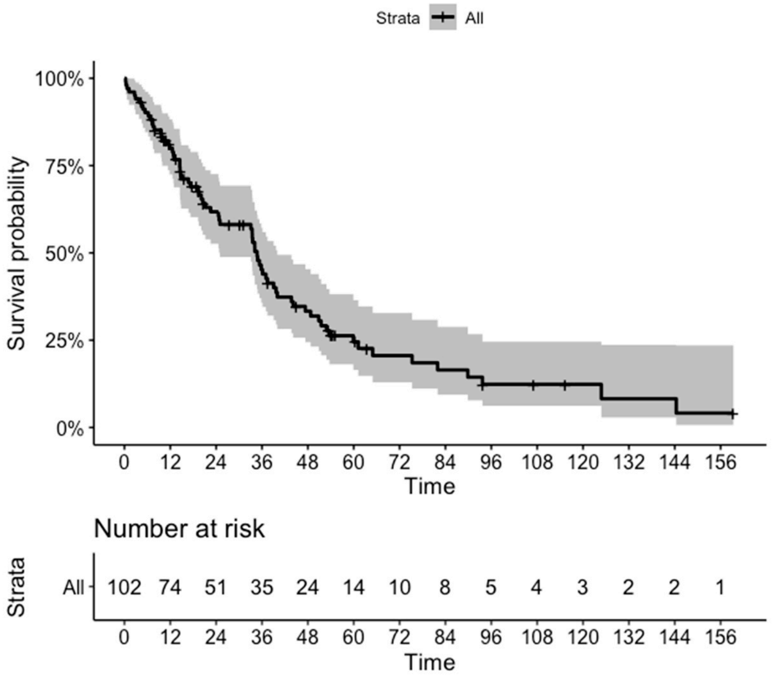

Supp Figure 1 : Overall survival from histological diagnosis in the whole population (N=102)

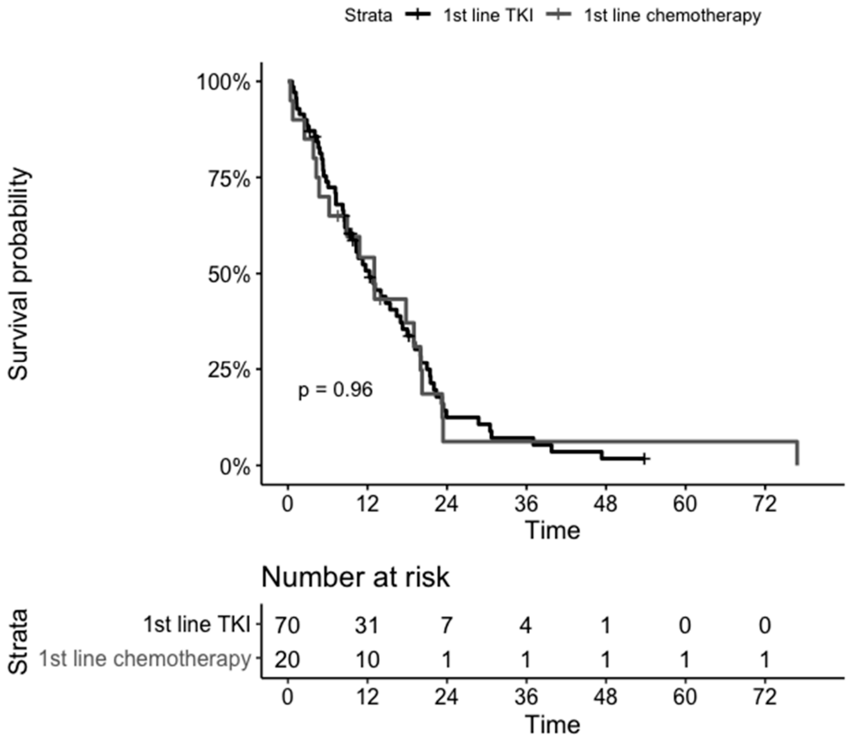

Supp Figure 2 : Progression-free survival (from the time of initiation of TKI therapy) in patients treated upfront with TKIs or in patients having received chemotherapy as a first line regimen.
